# Prioritizing Patient Selection in Clinical Trials: A Machine Learning Algorithm for Dynamic Prediction of In-Hospital Mortality for ICU Admitted Patients Using Repeated Measurement Data

**DOI:** 10.3390/jcm14020612

**Published:** 2025-01-18

**Authors:** Emma Pedarzani, Alberto Fogangolo, Ileana Baldi, Paola Berchialla, Ilaria Panzini, Mohd Rashid Khan, Giorgia Valpiani, Savino Spadaro, Dario Gregori, Danila Azzolina

**Affiliations:** 1 Clinical Trial and Biostatistics, Research and Innovation Unit, University Hospital of Ferrara, 44124 Ferrara, Italy; emma.pedarzani@ospfe.it (E.P.); ilaria.panzini@aospfe.it (I.P.); giorgia.valpiani@ospfe.it (G.V.); 2 Intensive Care Unit, University Hospital of Ferrara, 44124 Ferrara, Italy; alberto.fogagnolo@gmail.com (A.F.); savino.spadaro@unife.it (S.S.); 3 Unit of Biostatistics, Epidemiology and Public Health, Department of Cardiac, Thoracic and Vascular Sciences, University of Padua, 35131 Padua, Italy; ileana.baldi@unipd.it (I.B.); mohdrashid.khan@ubep.unipd.it (M.R.K.); dario.gregori@unipd.it (D.G.); 4 Department of Clinical and Biological Sciences, University of Turin, 10043 Turin, Italy; paola.berchialla@unito.it; 5 Department of Translational Medicine and for Romagna, University of Ferrara, 44124 Ferrara, Italy; 6 Department of Environmental Sciences and Prevention, University of Ferrara, 44124 Ferrara, Italy

**Keywords:** mortality prediction, machine learning, intensive care unit, clinical trial, patient recruitment

## Abstract

**Background:** A machine learning prognostic mortality scoring system was developed to address challenges in patient selection for clinical trials within the Intensive Care Unit (ICU) environment. The algorithm incorporates Red blood cell Distribution Width (RDW) data and other demographic characteristics to predict ICU mortality alongside existing ICU mortality scoring systems like Simplified Acute Physiology Score (SAPS). **Methods:** The developed algorithm, defined as a Mixed-effects logistic Random Forest for binary data (MixRFb), integrates a Random Forest (RF) classification with a mixed-effects model for binary outcomes, accounting for repeated measurement data. Performance comparisons were conducted with RF and the proposed MixRFb algorithms based solely on SAPS scoring, with additional evaluation using a descriptive receiver operating characteristic curve incorporating RDW’s predictive mortality ability. **Results:** MixRFb, incorporating RDW and other covariates, outperforms the SAPS-based variant, achieving an area under the curve of 0.882 compared to 0.814. Age and RDW were identified as the most significant predictors of ICU mortality, as reported by the variable importance plot analysis. **Conclusions**: The MixRFb algorithm demonstrates superior efficacy in predicting in-hospital mortality and identifies age and RDW as primary predictors. Implementation of this algorithm could facilitate patient selection for clinical trials, thereby improving trial outcomes and strengthening ethical standards. Future research should focus on enriching algorithm robustness, expanding its applicability across diverse clinical settings and patient demographics, and integrating additional predictive markers to improve patient selection capabilities.

## 1. Introduction

The Intensive Care Unit (ICU) is a complex environment where critically ill patients with severe, often life-threatening conditions are managed. In this setting, conducting Randomized Controlled Trials (RCTs) is particularly challenging due to the high variability in patient conditions, the urgency of interventions, and the ethical considerations inherent in studying critically ill populations [1]. An important aspect of successfully conducting ICU RCTs is identifying patients most likely to benefit from the intervention being studied [2]. ICU RCTs might face high mortality rates, leading to elevated dropout rates that compromise result validity. These patients typically suffer from life-threatening conditions such as sepsis, Acute Respiratory Distress Syndrome (ARDS), or Ventilator-Associated Pneumonia (VAP). Despite stringent inclusion/exclusion criteria, the presence of vague diagnoses often results in heterogeneous study populations for ICU RCTs [3]. François and colleagues discuss the recurring failures of ICU trials and emphasize the need for better patient stratification methods to improve trial outcomes [3]. In another study, Ali and colleagues highlight the difficulty of maintaining continuity of care in ICU settings and the impact this has on patient outcomes and trial integrity [4]. Clinicians conducting ICU trials face multiple obstacles, striving to balance advancing research while safeguarding participants from risks [4]. Ethical concerns, including withdrawal of care and obtaining consent from critically ill patients, further complicate trial conduct [5]. This complex framework highlights the need for a suitable and reliable mortality risk score that can guide patient selection in these clinical trials [6].

Moreover, a well-calibrated ICU-specific mortality risk score could significantly improve clinical trial design by identifying patients most likely to benefit from treatments. For instance, in sepsis trials, such a score could pinpoint patients with a higher chance of surviving long enough to experience the potential benefits of a novel therapy, thereby improving trial outcomes and patient selection [7]. Similarly, in ARDS trials, a dynamic mortality risk score can identify patients at critical moments when interventions might have the greatest impact, enabling targeted, timely treatments that could improve patient outcomes [8]. A suitable mortality risk score could also be important in trials for conditions like Acute Kidney Injury (AKI) or ventilator-associated pneumonia, as it ensures that those most likely to benefit are selected, optimizing resources and improving the ethical conduct of the trial [9].

Traditional ICU mortality scoring systems, such as the Acute Physiology and Chronic Health Evaluation (APACHE) [10], the Simplified Acute Physiology Score (SAPS) [11], and the Mortality Probability Models (MPMs) [12], have been extensively used to predict patient outcomes and guide clinical decision making. However, these scores have limitations, particularly when applied to the diverse and rapidly changing patient populations typically found in ICUs [13]. For example, while widely used, the APACHE score may not fully capture the dynamic nature of a patient’s condition over time, potentially leading to less accurate mortality predictions [14]. Moreover, for clinicians screening patients for clinical trials, a rapid and easy-to-use tool should be useful, as the time and resources required for detailed assessments can be significant barriers in fast-paced ICU settings [3]. Traditional scoring methods like SAPS [11] also rely on a large number of variables, including arterial blood gases and other complex physiological measurements, which may not always be readily available in real time. This complexity can impede the timely identification of eligible patients and potentially delay enrollment processes, particularly in resource-limited environments.

In recent years, there has been growing interest in integrating dynamic variables and Machine Learning (ML) approaches to improve mortality prediction in the ICU [15]. The ML models offer advantages over classical statistical models in handling complex, high-dimensional data typical of ICU environments, providing more accurate and dynamic predictions by capturing nonlinear relationships and interactions that traditional models may miss [16].

The application of advanced ML algorithms in this context is exemplified by several studies in the literature. For instance, Makino and colleagues developed an artificial intelligence model to predict the progression of diabetic kidney disease using big data, demonstrating the potential of ML in enhancing clinical decision making [17]. Similarly, Li et al. used a machine learning-based approach to predict in-hospital mortality in ICU patients with heart failure, further illustrating the value of ML in refining mortality risk prediction [18].

In this general framework, incorporating Red blood cell Distribution Width (RDW) into ML models as a predictive marker could represent an improvement in the field of ICU mortality prediction [19]. RDW is a measure of the variation in the size of red blood cells and is routinely obtained from standard complete blood count tests. RDW has been studied for its predictive ability regarding mortality and severe clinical outcomes [20,21,22]. Moreover, it is a cost-effective measure of anisocytosis, easily available through inexpensive blood tests, reflecting multiple acute and chronic conditions influencing mortality likelihood in ICUs [23,24]. While RDW could enhance predictive accuracy in ICU mortality, traditional tools reported in the literature using this marker [25] often fail to fully utilize ICU dynamic data over hospital stay [24,26].

This study presents an ML-based prognostic mortality scoring system that utilizes RDW and readily available patient characteristics in the ICU setting, incorporating repeated measurements to develop a user-friendly and easily accessible patient risk profiling tool for ICU use. The proposed Mixed-effects logistic Random Forest for binary data (MixRFb) algorithm combines the strengths of random forests and mixed-effects models to address the challenges posed by repeated measurements in ICU datasets. Traditional random forest models are well-suited for predicting clinical outcomes in ICU [27]; however, they assume independence among observations, which may lead to biased estimates in hierarchical data [28]. The mixed-effects component of the proposed MixRFb algorithm explicitly models intra-patient correlations by incorporating random effects, thereby capturing the variability associated with repeated measures such as RDW [29]. However, the mixed model alone has been deemed in several cases to have poor predictive performance on longitudinal data in comparison to machine learning models [29,30]. The MixRFb integration proposed in this research, instead, is aimed at addressing both fixed effects (consistent across patients) and random effects (individual-specific variations) components in combination with the improved predictive ability of an RF model [28]. Such an approach is particularly well-suited for ICU settings, where longitudinal clinical data play an important role in mortality prediction [31]. The predictive tool is designed to assist ICU patient selection while effectively managing repeated measurement data collected during the ICU stay.

## 2. Materials and Methods

### 2.1. Data

The dataset used for training the machine consists of 286 patients with at least 48 h of ICU length of stay who were hospitalized in the ICU of the University Hospital of Ferrara between August 2016 and December 2017. Clinical data were measured over five days of ICU stay, underlying their longitudinal nature.

Patients anticipated to have an ICU stay of at least 48 h were eligible for inclusion in the study. Exclusion criteria included individuals under 18 years of age, those with a history of hematological diseases, and pregnant women. Clinical and demographic data were collected daily. Written informed consent was obtained from all patients who were capable of providing it or from their next of kin [23].

The data collection was approved by the local Ethics Committee (CE AVEC) as reported by Fogagnolo et al. [23] with protocol number 160699 and a date of approval of 14 July 2016.

### 2.2. Study Size

Preliminary data analyses indicated that a sample size of approximately 280 patients would provide the necessary statistical power of 80% to detect a minimum significant difference in mortality prediction accuracy of 10% between the newly developed MixRFb model and existing models like SAPS, with a significance level set at 5%.

### 2.3. Descriptive Statistics

The absolute and relative frequencies according to the ICU mortality status are reported for the categorical variable and the median with interquartile ranges for the quantitative ones, together with the Odds Ratio (OR), 95% Confidence Intervals (CIs), and *p*-value for the univariable logistic regression model.

### 2.4. Machine Learning Models

Mixed effects Random Forest for binary data (MixRFb) is considered for developing the ML tool [32]. The algorithm combines the strengths of Random Forest (RF) and mixed-effects models for repeated measurement data. It first uses an RF-based algorithm to handle complex relationships in the data by creating multiple decision trees. Then, it incorporates a mixed-effects model to account for the correlation in repeated measurements, adjusting for both fixed effects (variables consistent across observations) and random effects (variables that vary). While traditional mixed-effects models account for hierarchical structures in data, they rely on predefined linear relationships and interaction terms, which may limit their flexibility in capturing complex patterns. The MixRFb model, in contrast, uses the random forest framework to automatically learn nonlinear relationships and higher-order interactions [33]. Technical details concerning the algorithm are reported in the Supplementary Material with Figure S1 reporting the algorithm flowchart.

#### Models Description

Several models have been trained and internally validated. During the procedure the the Multivariate Imputation by Chained Equations (MICEs) method, to address missing data [34], has been employed (see Section 2.5.2).

(1) Model A: MixRFb model using RDW as a predictor and also considering the following as features: (a) age, (b) gender, (c) time (days of ICU stay), (d) any comorbidity. The selection of covariates for the MixRFb algorithm was guided by their clinical relevance and availability in routine ICU practice. Age is widely recognized as a predictor of ICU mortality, capturing baseline patient severity and physiological derangements [35]. RDW was included as it reflects systemic inflammation and oxidative stress, which are critical in predicting outcomes in critically ill patients [26]. Comorbidities were considered to account for underlying health conditions that influence mortality risk [36]. In this study, we included the presence of at least one comorbidity, such as diabetes [37], cardiovascular disease [38], or respiratory disease [39], as these specific conditions are strongly associated with ICU mortality and provide clinically interpretable variables while simplifying data collection in high-pressure ICU environments. Gender was included to evaluate potential sex-related differences in outcomes, while days in ICU capture longitudinal changes in patient status [40].
(2) Model B: MixRFb model using SAPS as a predictor because it is widely recognized as a robust predictor of ICU mortality, capturing baseline patient severity and physiological derangements [41].
(3) Model C: Classical RF model using RDW as a predictor and also including the following as features: (a) age, (b) gender, (c) time (days of ICU stay), (d) any comorbidity.

### 2.5. Model Training Validation Workflow

**Figure 1:**
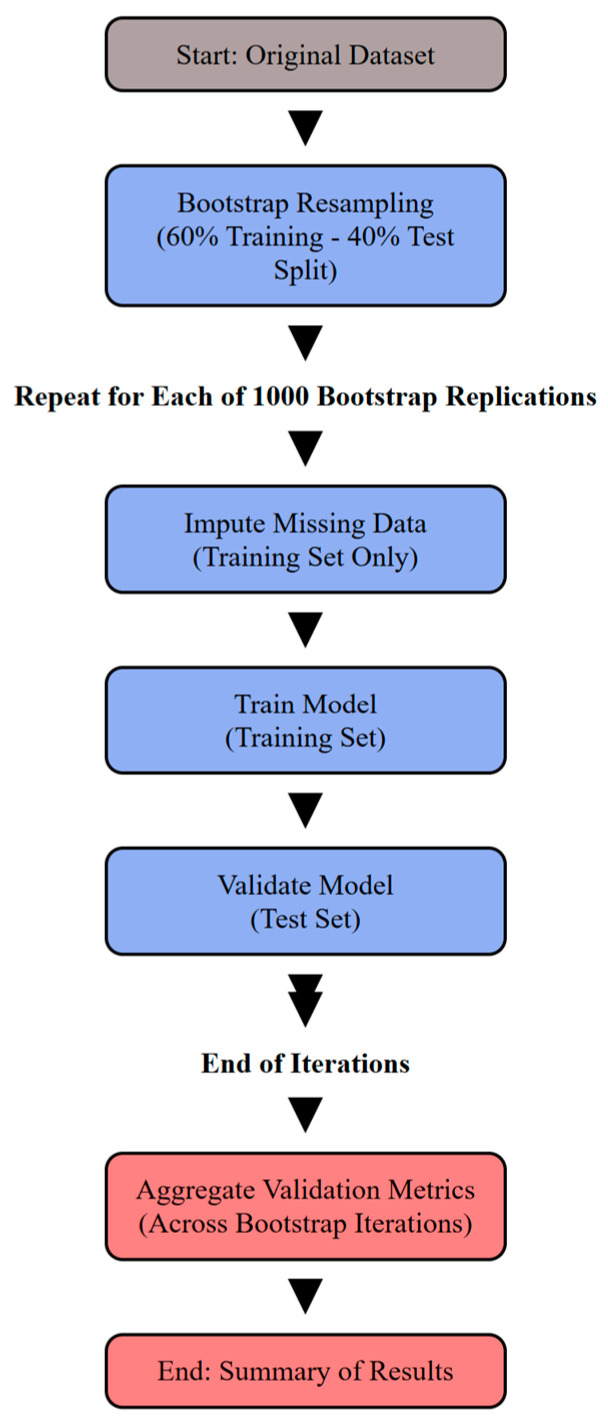
Model training and validation flowchart.

The flowchart in Figure 1 illustrates the workflow for training and validating machine learning models with bootstrap resampling. It begins with the original dataset, which undergoes bootstrap resampling to split it into training (60%) and testing (40%) sets. Each bootstrap iteration involves imputing missing data into the training set, training the model on the training data, and validating it on the test set. These steps are repeated for 1000 bootstrap replications. After completing all iterations, the aggregated validation metrics from all replications are computed, culminating in the final summary of results. Different colors highlight key stages: gray for initialization, blue for iterative steps, and red for post-validation aggregation and finalization.

Each step is further detailed in the subsequent paragraphs.

#### 2.5.1. Model Validation via Bootstrap Resampling

To assess and compare the performances, all models were internally validated in 1000 runs of bootstrap resampling.

For each of the 1000 bootstrap iterations, the model was trained on a resampled dataset (60% of the data) and evaluated on the Out-Of-Bag (OOB) observations not included in the bootstrap sample (Figure 1). This method ensures that each observation is used for both training and validation across iterations, providing reliable performance metrics while preserving the balance of mortality outcomes [42].

#### 2.5.2. Handling Missing Data

The study employed The MICE imputation [34]. The procedure imputes missing values iteratively while preserving the relationships between variables. This approach supports both continuous and categorical variables. Additionally, to mitigate the impact of imbalanced mortality outcomes, oversampling of the minority class (non-survivors) was performed during the training phase. Details concerning the distribution of missing data have been reported in the Supplementary Material.

The MICE imputation process was carried out within the validation phase in each bootstrap iteration on the training data only to prevent data leakage. After imputation, models were trained on the imputed training data, and predictions were validated on the corresponding test data (Figure 1).

#### 2.5.3. Measures of Performance

After completing all iterations, the aggregated validation metrics from all replications were computed. Performance metrics, including the F1-score, were computed to complement the AUC. The F1-score, the harmonic mean of precision and recall, provides a balanced evaluation of the model’s performance, particularly in imbalanced datasets where standard accuracy metrics may be misleading. The (a) training and (b) bootstrap validation performances, Area Under the ROC Curve (AUC), and F1 statistics have been reported for the trained models. The main model’s performance is compared with a descriptive mortality ROC analysis based on RDW and SAPS as variables.

### 2.6. Variable Importance

The Variable Importance Plot (VIP) has been reported, indicating the mean decrease in accuracy after each predictor is removed from the RF model component.

The algorithm’s predictor contributions were further evaluated using a multi-way importance plot. This visualization combines the Gini decrease in accuracy, a measure of a variable’s contribution to model performance, with the frequency of the variable’s inclusion in root nodes of decision trees, highlighting its influence on early splits. Additionally, *p*-values were calculated for each variable using a one-sided binomial test, based on the binomial distribution Bin (no of nodes, *P*(node splits on *X_j_*)), where *P*(node splits on *X_j_*) assumes that *X_j_* was uniformly drawn from the candidate variables. This analysis provides a statistical measure of the likelihood that each variable’s importance is greater than expected by chance.

Moreover, to improve interpretability, Partial Dependence Plots (PDPs) were generated for the leading predictors according to the multi-way importance analysis binomial test. PDPs illustrate each predictor’s marginal effect on the predicted mortality probabilities, holding other variables constant. This approach provides an intuitive understanding of the relationship between individual predictors and mortality risk.

To evaluate nonlinear relationships and interactions between predictors, the mean minimal depth of interactions measured within the MixRFb model was also analyzed. The results are presented in Supplementary Material.

### 2.7. Sensitivity Analyses

✓ Standalone Variable Predictive Analysis. The standalone predictive power of individual variables was evaluated by fitting a MixRFb model using variables selected based on their prominence in a multi-way importance analysis, which identified them as leading predictors. The analysis involved fitting the model for each variable independently to assess their predictive capability.
✓ Sensitivity Analysis with Recurrent Neural Network. A Recurrent Neural Network (RNN) was implemented as a sensitivity analysis to handle repeated measurement data via MLT. The model incorporated four features: age, gender, days in ICU, and RDW. The RNN was configured with a batch size of 286 and five time points with a discrete outcome.
✓ Sensitivity Analysis with Generalized Linear Mixed Effect Model. A sensitivity analysis with a simple mixed-effect model was also performed.
✓ Descriptive ROC Analysis. A descriptive Receiver Operating Characteristic (ROC) analysis was conducted to evaluate the performance of traditional SAPS and RDW as standalone predictors. ROC curves were generated for each variable independently to assess their predictive capacity.

The computation was carried out using R 4.3.1 [43], together with the caret [44] package.

### 2.8. Shiny Application Development

A Shiny application was created to make the MixRFb algorithm easily accessible for practitioners, enabling real-time prediction of ICU mortality risk.

## 3. Results

This study reports the baseline characteristics of 286 ICU patients, with 207 surviving and 79 dying in ICU. Table 1 displays these characteristics categorized by mortality. Statistically significant associations were detected for age, SAPS, and diabetes. Surviving patients tended to be younger, with lower SAPS scores, and without diabetes, indicating less severe acute illness at baseline. Gender, cardiovascular disease, respiratory disease, and comorbidity presence did not show significant associations with survival status (*p* > 0.05).

Table S1 (Supplementary Material) points out stable RDW values over time, with statistically significant differences observed at all time points (*p* < 0.001), except for urea. Other longitudinal clinical variables show no significant differences across days. The proportion of missing data in the variables used for training the algorithms is lower than 23% (Figure S2, Supplementary Material).

### 3.1. Model Performances

#### 3.1.1. Training

**Figure 2:**
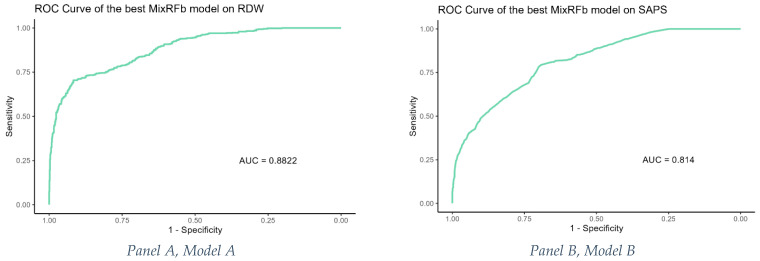
ROC curves for the MixRFb algorithm on RDW and other covariates (**Panel A**, **Model A**) and MixRFb on SAPS (**Panel B**, **Model B**) for the training performance.

✓ Model A (MixRFb incorporating RDW and other covariates): The training AUC was 0.882 (95% CI: 0.860–0.904), indicating a strong predictive performance. This result is graphically represented in Figure 2, Panel A.
✓ Model B (MixRFb using SAPS as a predictor): This model showed a reduced training performance, with an AUC of 0.814 (95% CI: 0.790–0.838), suggesting that while SAPS is a useful predictor, the addition of RDW and other covariates in Model A improves prediction accuracy.

The training performance of Model B is also depicted in Figure 1, Panel B.

✓ Model C (Classical RF using RDW as a predictor): Although not incorporating repeated measurement data, this model demonstrated a training AUC of 0.835 (95% CI: 0.812–0.858).

#### 3.1.2. Validation

Concerning the bootstrap validation procedure, Model A achieved a median F1-score of 0.76 (95% CI: 0.72–0.78) in addition to a median AUC of 0.87 (95% CI: 0.85–0.88). The metrics confirmed, also for the internal validation, the improved predictive ability of Model A in comparison with Model B and Model C (Table 2).

### 3.2. Variable Contribution

**Figure 3:**
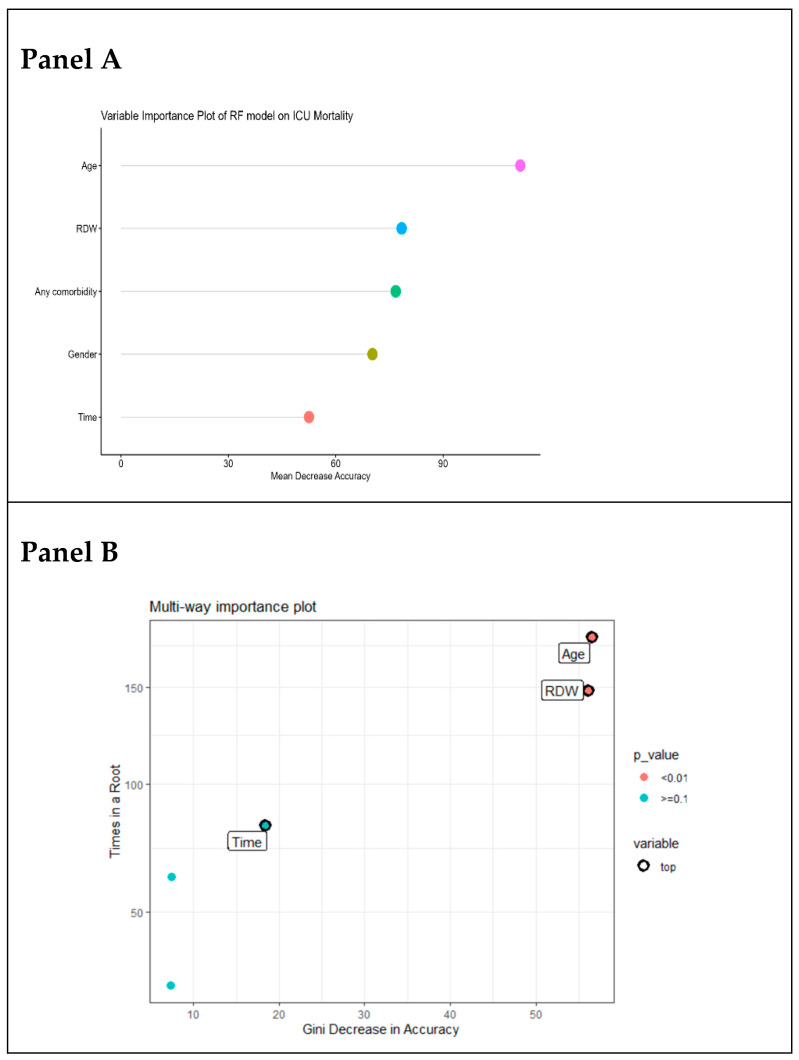
Variable importance plot for the mean decrease in accuracy of RF classification part (**Panel A**). Multi-way importance plot (**Panel B**). This visualization combines the Gini decrease in accuracy—a measure of a variable’s contribution to model performance—with the frequency of the variable’s inclusion in root nodes of decision trees, highlighting its influence on early splits.

The Variable Importance Plot (VIP) for the RF part of the algorithm, shown in Figure 3, emphasizes the significance of age and RDW in predicting ICU mortality (Panel A).

Moreover, the multi-way importance plot confirmed that RDW and age were the most significant predictors, with the highest Gini decreases in accuracy and frequent inclusion in root nodes. Time (days in ICU) also emerged as an important variable, reflecting the algorithm’s ability to capture temporal changes in patient status (Figure 3, Panel B).

**Figure 4:**
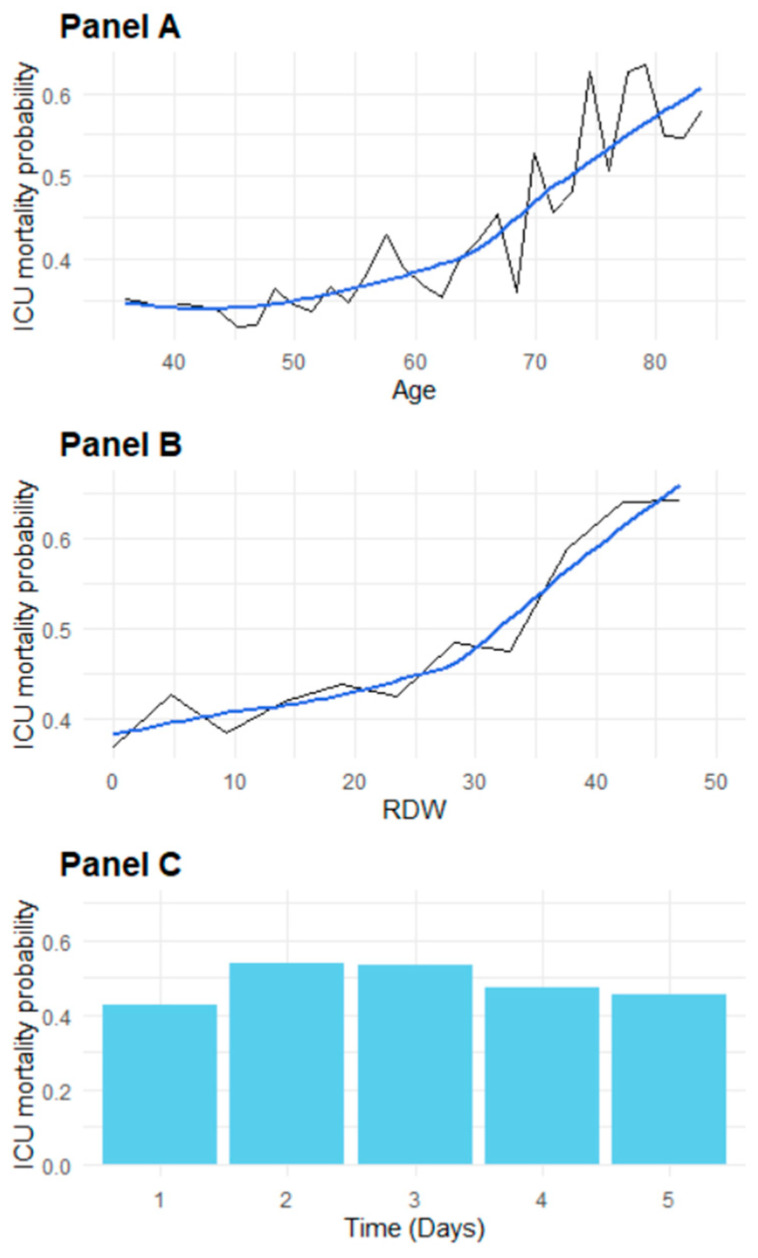
Partial Dependency Plots (PDPs) illustrating key predictors of ICU mortality risk. (**Panel A**) PDP showing the effect of age on the predicted probability of death; (**Panel B**) PDP depicting the relationship between RDW (Red cell Distribution Width) and predicted mortality risk. Panel A and B report both the predicted values in black and loess smoothed values in blue; (**Panel C**) PDP bar graph of predicted mortality risk over a consecutive five-day period.

The partial dependence plots (Figure 4) describe how age, RDW, and time influence mortality probabilities in the algorithm. The plot for age reveals a near-linear increase in mortality risk with advancing age (Panel A). For RDW, mortality risk rises sharply, particularly at values above 16% (Panel B). The plot for time shows relatively stable predicted probabilities across days, suggesting that patients who survive longer in the ICU may stabilize over time (Panel C).

The mean minimal depth of interactions within the MixRFb model is presented in Supplementary Material, Figure S3, identifying age and RDW as the most frequent and important interacting variables, with interactions consistently appearing near the root of the decision trees.

### 3.3. Sensitivity Analyses

*Standalone Variable Predictive Analysis.* To evaluate the standalone predictive power of individual variables, a MixRFb using only age and RDW has been estimated. These variables are leading predictors according to the multi-way importance analysis. The model using age alone achieved an AUC of 0.712 (95% CI: 0.687–0.734), demonstrating its moderate predictive ability. In contrast, RDW alone yielded an AUC of 0.61 (95% CI: 0.58–0.63).

*Sensitivity Analysis with Recurrent Neural Network.* The RNN model demonstrated a lower performance compared to the MixRFb algorithm, with an AUC score of 0.77 (95% CI: 0.75, 0.79) and an F1 score of 0.70 (95% CI: 0.68, 0.72). In contrast, the MixRFb algorithm achieved superior predictive performance, showcasing higher metrics across evaluation criteria.

*Sensitivity Analysis with Mixed Effect Model.* The AUC for the simple mixed model is lower than MixRFb and equal to 0.64 (95% CI: 0.4–0.74).

*Descriptive ROC Analysis.* The descriptive ROC curve has been calculated for the traditional SAPS and RDW:

✓ Using SAPS alone: The ROC curve analysis for SAPS as a standalone predictor displayed an AUC of 0.683 (95% CI: 0.655–0.711), underlining a weaker predictive capability.
✓ Using RDW alone: RDW’s predictive ability for mortality was the lowest, with an AUC of 0.555 (95% CI: 0.527–0.583), suggesting limited utility when used without modeling patterns and interaction with additional predictors.

### 3.4. Shiny App

The web-based predictive tool for the best-performing algorithm (Model A) is available online at (https://biostatlab24.shinyapps.io/MixRFbICU/, accessed on 17 January 2025).

The MixRFb algorithm, implemented via a Shiny application, is optimized for computational efficiency. Its reliance on the parallelizable random forest framework enables rapid predictions.

**Figure 5:**
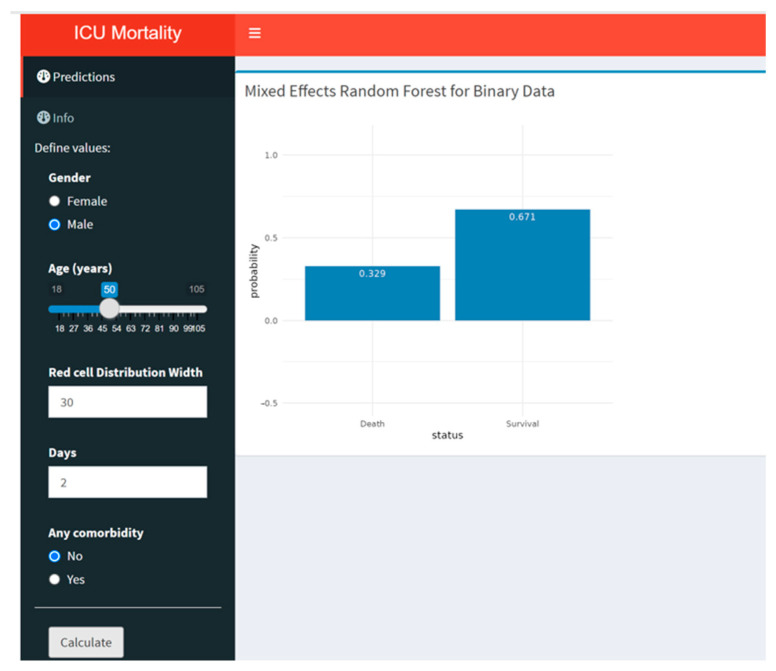
MixRFb online predictive tool.

This Shiny app is designed to predict ICU mortality using a MixRFb algorithm (Model A). Users can input patient data such as gender, age, RDW, days in ICU, and comorbidity status. After clicking “Calculate”, the app displays the predicted probabilities for death and survival in a bar chart, helping practitioners assess the patient’s risk in real time. The interface is simple, with inputs on the left and the prediction results visualized on the right (Figure 5).

## 4. Discussion

This study demonstrates the potential of the MixRFb algorithm in predicting in-hospital mortality using repeated measurement data, with performance comparable to or exceeding other ICU mortality algorithms in the literature [17,45].

The proposed algorithm demonstrates a slight improvement in performance for predicting ICU mortality in comparison with a simple RF version based on SAPS. The algorithm is developed using cost-effective and time-efficient parameters like RDW. The SAPS score offers a useful baseline assessment, but it cannot capture dynamic changes in a patient’s condition during their ICU stay. The RDW evolution, which could be handled in a MixRFb model, in contrast, reflects systemic inflammation, oxidative stress, and other evolving physiological processes, making it a powerful and accessible biomarker for longitudinal risk assessment [46]. For example, two patients with similar SAPS scores might exhibit different trajectories during their ICU stay. A rising RDW in one patient could signal worsening inflammation or complications such as sepsis, a critical insight missed by SAPS [47]. Moreover, SAPS, while widely used for mortality prediction, relies on an extensive list of physiological, laboratory, and clinical variables, many of which require time-intensive or resource-dependent data collection [41]. This complexity can limit its utility in real-time decision-making, particularly for identifying eligible participants for clinical trials. By contrast, the MixRFb algorithm uses just a few variables that are readily available in routine clinical practice. This simplicity allows for faster and more practical stratification of patients, making it easier to identify participants for trial inclusion.

The integration of mixed-effects modeling within the RF framework provides several advantages in the analysis of ICU datasets. ICU data are inherently hierarchical, with repeated measurements collected for each patient over time. The mixed-effects component allows for the explicit modeling of intra-patient correlations, addressing potential biases arising from the dependence on observations [31]. The MixRFb algorithm improves the predictive accuracy of the model, outperforming the classical mixed effect model in terms of predictive ability as indicated in the literature and also accounting for our results. The algorithm’s ability to capture nonlinear relationships and higher-order interactions allows it to flexibly model complex data structures without requiring explicit specification of interactions or transformations [28]. The algorithm’s superior performance of Model A suggests that it can better capture the complex, nonlinear relationships within ICU data, which is important for an accurate mortality prediction [2].

The inclusion of longitudinal data, such as days in ICU, allows the model to capture temporal changes in patient status, addressing a critical limitation of static scoring systems [36]. Additionally, the covariates included in the algorithm were selected to balance the need for predictive power, clinical relevance, and practical applicability, facilitating the development of a tool that remains both robust and deployable in diverse ICU settings [48].

Comorbidities and gender, though not always individually significant, contribute to a holistic risk assessment by accounting for variability in patient characteristics and outcomes [40]. The standalone performance of age highlights its strong individual contribution to ICU mortality prediction. Age and RDW emerged as primary predictors, consistent with their critical role in assessing patient vulnerability and health status [23]. The RDW, in particular, is an important clinical predictor, validating its relevance in critical care settings and improving the model’s predictive capacity [23]. Beyond predictive accuracy, RDW’s routine availability as part of a complete blood count improved its practicality. This accessibility makes the MixRFb model an easy tool relevant for ICU settings, where rapid and actionable insights are important [14,23,24]. Age is important for understanding patient severity at admission [35], while RDW adds value as a dynamic biomarker indicative of systemic inflammation and acute stress [26].

However, while age performs well independently, it lacks the interactions captured by the MixRFb model, which combines multiple variables such as RDW, days in ICU, and comorbidities. Similarly, RDW, while limited as a standalone predictor, becomes more influential when combined with other variables, reflecting its role as a marker of systemic inflammation that complements age and other clinical factors. For example, the interaction between age and RDW, identified as highly influential, suggests that the combined effects of age-related vulnerability and systemic inflammation are greater than the sum of their contributions to mortality risk [49].

The limited standalone importance of RDW does not diminish its clinical relevance; the PDP analysis reveals an association between higher RDW values and increased mortality, reflecting its role as a marker of systemic inflammation and critical illness severity [50]. Similarly, the consistent rise in mortality risk with age underscores its importance in risk stratification. These findings reinforce the importance of using integrated predictive models rather than relying on single-variable analyses, especially in critical care settings where outcomes are influenced by complex and interrelated factors [51].

Moreover, the superior performance of the MixRFb over another widely used algorithm, the RNN model, underscores its ability to capture some variable relations of ICU data, which are not as effectively addressed by the RNN in this specific situation. RNN is a highly performing algorithm but may require larger datasets or a higher resolution of temporal features to fully utilize its sequential modeling capabilities [52]. In contrast, the MixRFb model is specifically tailored to the given ICU context.

The MixRFb tool could assist multicenter ICU trials by standardizing patient selection across diverse sites, thus reducing variability and improving trial reliability, particularly in cases like AKI disease where patient homogeneity is particularly relevant [9]. Otherwise, during ICU overcrowding, such as during pandemics, the tool could aid in efficient resource allocation by drawing attention to patients who would benefit most from intensive treatments, ensuring the optimal use of limited resources while supporting ethical decisions [19]. For patients with chronic conditions like COPD or heart failure, the tool facilitates tailored interventions by predicting outcomes based on repeated clinical measurements, potentially improving patient recovery and reducing ICU stay [17]. Additionally, the tool’s predictive capabilities extend into post-ICU care, guiding decisions on the level of care needed after discharge and helping to prevent readmissions; for example, patients identified as having a high risk of post-ICU mortality might be candidates for closer monitoring in a step-down unit or for more intensive rehabilitation services [21].

Aligning with precision medicine goals, the tool customizes treatment strategies based on individual patient profiles, improving the effectiveness of the trial design and increasing the likelihood of successful outcomes in severe ill conditions such as sepsis.

Furthermore, the practical implementation of this algorithm through a user-friendly Shiny application facilitates its adoption in clinical settings, enabling practitioners to make informed decisions based on real-time predictions. This tool bridges the gap between advanced ML techniques and their application in everyday clinical practice, making predictive analytics accessible to healthcare providers at the bedside [18].

### Study Limitations and Future Research Developments

Challenges in data collection or quality may affect predictive accuracy, necessitating future research to externally validate the algorithm’s robustness and expand its applicability across clinical settings and patient characteristics [45]. For example, as RDW emerged as a significant predictor in our model, its limitations and potential biases must be acknowledged. As a routinely measured and cost-effective biomarker, RDW is useful, but variability across laboratories may affect its consistency and generalizability [53]. In diverse ICU settings, variability in laboratory standards, data collection protocols, and the completeness of medical records may challenge the model’s generalizability [50]. For instance, RDW measurements can vary depending on the specific equipment and methodology used. Standardizing data collection processes, such as ensuring uniform RDW measurement protocols, would further improve its robustness. Furthermore, future studies could adopt the algorithm to incorporate local calibration mechanisms, enabling it to adjust to site-specific data patterns while maintaining its predictive accuracy. Despite these limitations, RDW’s accessibility underscores its practical utility, making it a promising feature for improving predictive analytics while highlighting areas for further research and refinement [54].

The MixRFb algorithm’s computational efficiency allows it to operate seamlessly in real-time ICU environments, generating predictions quickly using standard hardware. Scalability is further supported by the web application, enabling ease of deployment in diverse settings. However, successful implementation will depend on real-time access to clinical data and robust integration with EHR systems. Cloud-based solutions may further improve scalability and accessibility, particularly in high-volume or resource-limited ICUs.

While the MixRFb algorithm demonstrates superior performance compared to SAPS, we acknowledge that the study’s comparison is limited to this single scoring system. Other established ICU scoring systems, such as the Acute Physiology and Chronic Health Evaluation (APACHE) [55] and Mortality Probability Models (MPM) [12], are also widely used for mortality prediction in critical care settings. These models, however, share limitations similar to SAPS, including reliance on static admission data and limited integration of longitudinal variables. Future studies should evaluate the algorithm against APACHE and MPM to provide a more extensive comparison and further validate its applicability across diverse ICU contexts.

Moreover, while internal validation through bootstrapping demonstrates the model’s robustness, external validation using independent datasets is important to assess the model’s generalizability across different ICU settings and patient populations. Future external validation studies are needed to confirm the model’s reliability and applicability beyond the development setting.

Additionally, the proposed algorithm provides useful predictive results; however, it does not replace clinical judgment but rather serves as a tool to assist healthcare providers in making more informed decisions. Ethical considerations must guide its use, ensuring that the algorithm complements, rather than overrides, the decision-making process of experienced clinicians.

## 5. Conclusions

This study highlights the MixRFb algorithm’s potential to improve ICU mortality prediction by incorporating dynamic data, especially the cost-effective and easily accessible RDW. The algorithm outperformed traditional models, including the SAPS score, demonstrating its superior ability to handle complex ICU data. The development of a Shiny application makes this tool practical for real-time clinical use, improving patient selection for trials, optimizing resource use in overcrowded ICUs, and supporting personalized treatment strategies. Future research should focus on validating and expanding the algorithm’s application across various settings to further improve ICU care and clinical trial design.

## Figures and Tables

**Table 1:** Baseline variables and repeated clinic measurements of ICU patients (N = 286).

Variables	Survival *(n = 207)*	Death *(n = 79)*	Total *(N = 286)*	OR	CI 95%	*p*-Value
Age (years) (median [IQR])	69.0 [59.0–78.0]	74.0 [66.0–79.0]	71.0 [61.0–78.0]	1.03	[1.00; 1.05]	0.022
Gender, n (%)				1.27	[0.71; 2.25]	0.496
Male (Ref.)	117 (63.9%)	39 (58.2%)	156 (62.4%)			
Female	66 (36.1%)	28 (41.8%)	94 (37.6%)			
Diabetes, n (%)				1.89	[1.04; 3.42]	0.046
No (Ref.)	135 (73.8%)	40 (59.7%)	175 (70.0%)			
Yes	48 (26.2%)	27 (40.3%)	75 (30.0%)			
Cardiovascular disease, n (%)				1.59	[0.91; 2.84]	
No (Ref.)	95 (51.9%)	27 (40.3%)	122 (48.8%)			0.138
Yes	88 (48.1%)	40 (59.7%)	128 (51.2%)			
Respiratory disease, n (%)				0.92	[0.44; 1.84]	
No (Ref.)	145 (79.2%)	54 (80.6%)	199 (79.6%)			0.953
Yes	38 (20.8%)	13 (19.4%)	51 (20.4%)			
SAPS (median [IQR])	36.0 [27.0; 46.0]	46.5 [39.0; 54.0]	40.0 [29.0; 50.0]	1.05	[1.03; 1.08]	<0.001
Any comorbidity, n (%)				1.56	[0.85; 2.97]	0.197
No (Ref.)	67 (36.6%)	18 (26.9%)	85 (34.0%)			
Yes	116 (63.4%)	49 (73.1%)	165 (66.0%)			

**Table 2:** AUC and F1 metrics were achieved via bootstrap validation. The 95% confidence intervals were calculated within 1000 iterations.

	AUC	F1
Model A	0.87 [0.85–0.88]	0.76 [0.72–0.78]
Model B	0.8 [0.79–0.83]	0.72 [0.69–0.74]
Model C	0.78 [0.8–0.81]	0.66 [0.72–0.77]

## Data Availability

The datasets used and/or analyzed during the current study are available from the corresponding author upon reasonable request.

## Supplementary Materials

The following supporting information can be downloaded at: https://www.mdpi.com/article/10.3390/jcm14020612/s1, Figure S1: Flowchart of MixRFb algorithm; Figure S2: Missing Data Plot. Proportion of missing data for features in the dataset, categorized by the total percentage of missing rows for each feature. The *y*-axis lists the features, relabeled as “Time”, “Age”, “Any Comorbidity”, “Gender”, and “RDW” for clarity. The *x*-axis shows the number of missing rows, with labels indicating the corresponding percentage of missing values for each feature. Features are grouped into bands based on the percentage of missing data, with “Good” (green) representing features with minimal missing data and “OK” (orange) for features with a higher proportion of missing values.; Figure S3: Mean Minimal Depth interaction plot. Mean minimal depth for the ten most frequent variable interactions identified in the random forest model. The *x*-axis represents variable interactions, while the y-axis indicates the mean minimal depth, which measures how early a variable interaction is utilized in the decision trees. Lower minimal depth reflects higher importance, as the interaction is used earlier in the model’s decision-making process. The red horizontal line represents a predefined threshold for minimal depth, with interactions below the line considered highly significant. The bars are colored to indicate the frequency of the interaction’s occurrence across the forest, with darker colors denoting higher frequencies. Black dots represent the unconditional importance of each interaction, calculated as the average impact of the interaction across the entire forest, independent of specific conditions. Error bars around the black dots indicate variability in unconditional importance, providing insight into the stability and consistency of each interaction’s significance. Table S1: Longitudinal clinical characteristics of ICU patients. References [56,57] are cited in the Supplementary Materials.

## Abbreviations

ICU	Intensive Care Unit
RCTs	Randomized Controlled Trials
ARDS	Acute Respiratory Distress Syndrome
VAP	Ventilator-Associated Pneumonia
AKI	Acute Kidney Injury
APACHE	Acute Physiology and Chronic Health Evaluation Score
SAPS	Simplified Acute Physiology Score
MPM	Mortality Probability Models
ML	Machine Learning
RDW	Red Blood Cell Distribution Width
OR	Odds Ratio
CI	Confidence Intervals
MixRFb	Mixed Effects Random Forest for Binary Data
RF	Random Forest
MICE	Missing Imputation Chain
AUC	Area Under ROC Curve
OOB	Out-Of-Bag
VIP	Variable Importance Plot

## References

[1] Delaney A., Angus D.C., Bellomo R., Cameron P., Cooper D.J., Finfer S., Harrison D.A., Huang D.T., Myburgh J.A., Peake S.L. (2008). Bench-to-Bedside Review: The Evaluation of Complex Interventions in Critical Care. Crit. Care.

[2] Granholm A., Alhazzani W., Derde L.P.G., Angus D.C., Zampieri F.G., Hammond N.E., Sweeney R.M., Myatra S.N., Azoulay E., Rowan K. (2022). Randomised Clinical Trials in Critical Care: Past, Present and Future. Intensive Care Med..

[3] François B., Clavel M., Vignon P., Laterre P.-F. (2016). Perspective on Optimizing Clinical Trials in Critical Care: How to Puzzle out Recurrent Failures. J. Intensive Care.

[4] Ali N.A., Wolf K.M., Hammersley J., Hoffmann S.P., O’Brien J.M., Phillips G.S., Rashkin M., Warren E., Garland A., on behalf of the Midwest Critical Care Consortium Continuity of Care in Intensive Care Units (2011). A Cluster-Randomized Trial of Intensivist Staffing. Am. J. Respir. Crit. Care Med..

[5] Luce J.M., Cook D.J., Martin T.R., Angus D.C., A Boushey H., Curtis J.R., E Heffner J., Lanken P.N., Levy M.M., Polite P.Y. (2004). The Ethical Conduct of Clinical Research Involving Critically Ill Patients in the United States and Canada: Principles and Recommendations. Am. J. Respir. Crit. Care Med..

[6] Yamga E., Mantena S., Rosen D., Bucholz E.M., Yeh R.W., Celi L.A., Ustun B., Butala N.M. (2023). Optimized Risk Score to Predict Mortality in Patients with Cardiogenic Shock in the Cardiac Intensive Care Unit. J. Am. Hear. Assoc..

[7] Boussina A., Shashikumar S.P., Malhotra A., Owens R.L., El-Kareh R., Longhurst C.A., Quintero K., Donahue A., Chan T.C., Nemati S. (2024). Impact of a Deep Learning Sepsis Prediction Model on Quality of Care and Survival. Npj Digit. Med..

[8] Villar J., Ferrando C., Tusman G., Berra L., Rodríguez-Suárez P., Suárez-Sipmann F. (2021). Unsuccessful and Successful Clinical Trials in Acute Respiratory Distress Syndrome: Addressing Physiology-Based Gaps. Front. Physiol..

[9] Koshiaris C., Archer L., Lay-Flurrie S., Snell K.I., Riley R.D., Stevens R., Banerjee A., Usher-Smith J.A., Clegg A., Payne R.A. (2023). Predicting the Risk of Acute Kidney Injury in Primary Care: Derivation and Validation of STRATIFY-AKI. Br. J. Gen. Pr..

[10] Knaus W.A., Zimmerman J.E., Wagner D.P., Draper E.A., Lawrence D.E. (1981). APACHE—Acute Physiology and Chronic Health Evaluation: A Physiologically Based Classification System: *Crit*. Care Med..

[11] Gall J.-R.L., Loirat P., Alperovitch A., Glaser P., Granthil C., Mathieu D., Mercier P., Thomas R., Villers D. (1984). A Simplified Acute Physiology Score for ICU Patients. Crit. Care Med..

[12] Lemeshow S. (1993). Mortality Probability Models (MPM II) Based on an International Cohort of Intensive Care Unit Patients. JAMA.

[13] Zhai Q., Lin Z., Ge H., Liang Y., Li N., Ma Q., Ye C. (2020). Using Machine Learning Tools to Predict Outcomes for Emergency Department Intensive Care Unit Patients. Sci. Re.p.

[14] Tian Y., Yao Y., Zhou J., Diao X., Chen H., Cai K., Ma X., Wang S. (2022). Dynamic APACHE II Score to Predict the Outcome of Intensive Care Unit Patients. Front. Med..

[15] Atallah L., Nabian M., Brochini L., Amelung P.J. (2023). Machine Learning for Benchmarking Critical Care Outcomes. Healthc. Inform. Res..

[16] Sikora A., Zhang T., Murphy D.J., Smith S.E., Murray B., Kamaleswaran R., Chen X., Buckley M.S., Rowe S., Devlin J.W. (2023). Machine Learning vs. Traditional Regression Analysis for Fluid Overload Prediction in the ICU. Sci. Rep..

[17] Makino M., Yoshimoto R., Ono M., Itoko T., Katsuki T., Koseki A., Kudo M., Haida K., Kuroda J., Yanagiya R. (2019). Artificial Intelligence Predicts the Progression of Diabetic Kidney Disease Using Big Data Machine Learning. Sci. Rep..

[18] Chen Z., Li T., Guo S., Zeng D., Wang K. (2023). Machine Learning-Based in-Hospital Mortality Risk Prediction Tool for Intensive Care Unit Patients with Heart Failure. Front. Cardiovasc. Med..

[19] Hong W.S., Rudas A., Bell E.J., Chiang J.N. (2023). Association of Red Blood Cell Distribution Width with Hospital Admission and In-Hospital Mortality Across All-Cause Adult Emergency Department Visits. JAMIA Open.

[20] Fontana V., Spadaro S., Villois P., Righy Shinotsuka C., Fogagnolo A., Nobile L., Vincent J.-L., Creteur J., Taccone F.S. (2018). Can Red Blood Cell Distribution Width Predict Outcome After Cardiac Arrest?. Minerva Anestesiol..

[21] Yonemoto S., Hamano T., Fujii N., Shimada K., Yamaguchi S., Matsumoto A., Kubota K., Hashimoto N., Oka T., Senda M. (2018). Red Cell Distribution Width and Renal Outcome in Patients with Non-Dialysis-Dependent Chronic Kidney Disease. PLoS ONE.

[22] Valko L., Baglyas S., Podmaniczky E., Prohaszka Z., Gal J., Lorx A. (2022). Exploring Red Cell Distribution Width as a Biomarker for Treatment Efficacy in Home Mechanical Ventilation. BMC Pulm. Med..

[23] Fogagnolo A., Spadaro S., Taccone F.S., Ragazzi R., Romanello A., Fanni A., Marangoni E., Franchi F., Scolletta S., Volta C.A. (2019). The Prognostic Role of Red Blood Cell Distribution Width in Transfused and Non-Transfused Critically Ill Patients. Minerva Anestesiol..

[24] Valenti A.C., Vitolo M., Imberti J.F., Malavasi V.L., Boriani G. (2021). Red Cell Distribution Width: A Routinely Available Biomarker with Important Clinical Implications in Patients with Atrial Fibrillation. CPD.

[25] She Y., Li Y., Chen S., Chen Y., Zhou L. (2022). Red Blood Cell Distribution Width Predicts In-Hospital Mortality in Patients with a Primary Diagnosis of Seizures in the ICU: A Retrospective Database Study. Neurol. Sci..

[26] Horne B.D., Anderson J.L., Muhlestein J.B., Ridker P.M., Paynter N.P. (2015). Complete Blood Count Risk Score and Its Components, Including RDW, Are Associated with Mortality in the JUPITER Trial. Eur. J. Prev. Cardiolog..

[27] Lee J. (2017). Patient-Specific Predictive Modeling Using Random Forests: An Observational Study for the Critically Ill. JMIR Med. Inform..

[28] Hu J., Szymczak S. (2023). A Review on Longitudinal Data Analysis with Random Forest. Brief. Bioinform..

[29] Dankl D., Rezar R., Mamandipoor B., Zhou Z., Wernly S., Wernly B., Osmani V. (2022). Red Cell Distribution Width Is Independently Associated with Mortality in Sepsis. Med. Princ. Pr..

[30] Hu S., Wang Y.-G., Drovandi C., Cao T. (2023). Predictions of Machine Learning with Mixed-Effects in Analyzing Longitudinal Data Under Model Misspecification. Stat. Methods Appl..

[31] Chen A., Zhao Z., Hou W., Singer A.J., Li H., Duong T.Q. (2021). Time-to-Death Longitudinal Characterization of Clinical Variables and Longitudinal Prediction of Mortality in COVID-19 Patients: A Two-Center Study. Front. Med..

[32] Wang J., Gamazon E.R., Pierce B.L., Stranger B.E., Im H.K., Gibbons R.D., Cox N.J., Nicolae D.L., Chen L.S. (2016). Imputing Gene Expression in Uncollected Tissues Within and Beyond GTEx. Am. J. Hum. Genet..

[33] Liaw A., Wiener M. (2002). Classification and Regression by random forest. R News.

[34] van Buuren S., Groothuis-Oudshoorn K. (2011). Mice: Multivariate Imputation by Chained Equations in R. J. Stat. Softw..

[35] Gonçalves-Pereira J., Oliveira A., Vieira T., Rodrigues A.R., Pinto M.J., Pipa S., Martinho A., Ribeiro S., Paiva J.-A. (2023). Critically Ill Patient Mortality by Age: Long-Term Follow-up (CIMbA-LT). Ann. Intensive Care.

[36] Forte J.C., Van Der Horst I.C.C. (2019). Comorbidities and Medical History Essential for Mortality Prediction in Critically Ill Patients. Lancet Digit. Health.

[37] Siegelaar S.E., Hickmann M., Hoekstra J.B., Holleman F., DeVries J.H. (2011). The Effect of Diabetes on Mortality in Critically Ill Patients: A Systematic Review and Meta-Analysis. Crit. Care.

[38] Rocha B., Cunha G., Maltes S., Moura A.N.N.E., Coelho F., Torres J., Santos P., Monteiro F., Monteiro F., Almeida G. (2021). Cardiovascular Disease in an Intensive Care Unit: Patterns of an Often Fatal Omen. Eur. Heart J..

[39] Grangier B., Vacheron C.-H., De Marignan D., Casalegno J.-S., Couray-Targe S., Bestion A., Ader F., Richard J.-C., Frobert E., Argaud L. (2024). Comparison of Mortality and Outcomes of Four Respiratory Viruses in the Intensive Care Unit: A Multicenter Retrospective Study. Sci. Rep..

[40] Merdji H., Long M.T., Ostermann M., Herridge M., Myatra S.N., De Rosa S., Metaxa V., Kotfis K., Robba C., De Jong A. (2023). Sex and Gender Differences in Intensive Care Medicine. Intensive Care Med..

[41] Le Gall J.-R. (1993). A New Simplified Acute Physiology Score (SAPS II) Based on a European/North American Multicenter Study. JAMA J. Am. Med. Assoc..

[42] Lin Z., Lawrence W.R., Huang Y., Lin Q., Gao Y. (2021). Classifying Depression Using Blood Biomarkers: A Large Population Study. J. Psychiatr. Res..

[43] R Core Team (2023). R: A Language and Environment for Statistical Computing.

[44] Kuhn M. (2008). Building Predictive Models in R Using the Caret Package. J. Stat. Softw..

[45] Li F., Xin H., Zhang J., Fu M., Zhou J., Lian Z. (2021). Prediction Model of In-Hospital Mortality in Intensive Care Unit Patients with Heart Failure: Machine Learning-Based, Retrospective Analysis of the MIMIC-III Database. BMJ Open.

[46] Moreno-Torres V., Royuela A., Múñez-Rubio E., Gutierrez-Rojas Á., Mills-Sánchez P., Ortega A., Tejado-Bravo S., García-Sanz J., Muñoz-Serrano A., Calderón-Parra J. (2022). Red Blood Cell Distribution Width as Prognostic Factor in Sepsis: A New Use for a Classical Parameter. J. Crit. Care.

[47] Duggal A., Scheraga R., Sacha G.L., Wang X., Huang S., Krishnan S., Siuba M.T., Torbic H., Dugar S., Mucha S. (2024). Forecasting Disease Trajectories in Critical Illness: Comparison of Probabilistic Dynamic Systems to Static Models to Predict Patient Status in the Intensive Care Unit. BMJ Open.

[48] Vallet H., Guidet B., Boumendil A., De Lange D.W., Leaver S., Szczeklik W., Jung C., Sviri S., Beil M., Flaatten H. (2023). The Impact of Age-Related Syndromes on ICU Process and Outcomes in Very Old Patients. Ann. Intensive Care.

[49] Said A.S., Spinella P.C., Hartman M.E., Steffen K.M., Jackups R., Holubkov R., Wallendorf M., Doctor A. (2017). RBC Distribution Width: Biomarker for Red Cell Dysfunction and Critical Illness Outcome?. Pediatr. Crit. Care Med..

[50] Peng S., Li W., Ke W. (2023). Association between Red Blood Cell Distribution Width and All-Cause Mortality in Unselected Critically Ill Patients: Analysis of the MIMIC-III Database. Front. Med..

[51] Stewart J., Bradley J., Smith S., McPeake J., Walsh T., Haines K., Leggett N., Hart N., McAuley D. (2023). Do Critical Illness Survivors with Multimorbidity Need a Different Model of Care?. Crit. Care.

[52] Waqas M., Humphries U.W. (2024). A Critical Review of RNN and LSTM Variants in Hydrological Time Series Predictions. MethodsX.

[53] Li N., Zhou H., Tang Q. (2017). Red Blood Cell Distribution Width: A Novel Predictive Indicator for Cardiovascular and Cerebrovascular Diseases. Dis. Markers.

[54] Zhang Z., Chew G.M., Shikuma C.M., Gangcuangco L.M.A., Souza S.A., Shiramizu B., Nakamoto B.K., Gong T., Mannem S.R., Mitchell B.I. (2018). Red Blood Cell Distribution Width as an Easily Measurable Biomarker of Persistent Inflammation and T Cell Dysregulation in Antiretrovirally Treated HIV-Infected Adults. HIV Clin. Trials.

[55] Knaus W.A., Draper E.A., Wagner D.P., Zimmerman J.E. (1985). APACHE II: A Severity of Disease Classification System. Crit. Care Med..

[56] Hothorn T., Hornik K., Zeileis A. (2006). Unbiased Recursive Partitioning: A Conditional Inference Framework. J. Comput. Graph. Stat..

[57] Xia R. (2009). Comparison of Random Forests and Cforest: Variable Importance Measures and Prediction Accuracies. Master’s Thesis.

