# Supplementary material for: Prioritizing Patient Selection in Clinical Trials: A Machine Learning Algorithm for Dynamic Prediction of In-Hospital Mortality for ICU Admitted Patients Using Repeated Measurement Data
Source: J Clin Med. 2025 Jan 18;14(2):612. doi: 10.3390/jcm14020612 (PMC11766334; doi:10.3390/jcm14020612)
Supplement: Supplementary file 1 [file jcm-14-00612-s001.zip › jcm-3320570-supplementary.pdf]

Supplementary Material

Compared to the original approach as proposed by Wang J. et al. (2016), which considers a Conditional Forest (CF) [57] as the base learner, our Mixed Effects Random Forest for Binary Data (MixRFb) in Model A and B employs the RF which is based on Breiman's RF algorithm for classification and regression.

The decision to use Breiman's RF algorithm in MixRFb, rather than the CF function, is supported by its proven ability to manage high-dimensional data and interactions without requiring extensive tuning, as demonstrated in studies where Random Forest outperformed other methods in predictive accuracy and stability across diverse datasets [58]. This makes it particularly well-suited for complex ICU data where precision is critical.

Finally, the MixRFb implemented algorithm has been applied to the ICU dataset for profiling the intrahospital mortality outcome.

The main statistical model built can be written as follows:

$$logit(Y_{ij}) = \mu_j + \beta_0 + \beta_1 gender_i + \beta_2 age_{ij} + \beta_3 any\ comorbidity_i + \beta_4 RDW_{ij} + \beta_5 time_j + \gamma_i + \epsilon_{ij}$$

- The $logit(Y_{ij})$ function is the natural logarithm of the odds of the dependent variable Y_{ij}. It transforms probabilities (ranging between 0 and 1).
- Y_{ij} is the dependent variable, indicating the outcome for individual *i* at time *j*.
- μ_j represents the fixed effect at time point j. It captures the baseline log-odds specific to each time point.
- β_0 is the intercept term, representing the baseline log-odds when all the other predictors are zero.
- β_1 the coefficient for the variable gender.
- β_2 is the coefficient for the variable age.
- β_3 is the coefficient for the variable any comorbidity.
- β_4 is the coefficient for the RDW measurement for individual i at time point j.
- β_5 is the coefficient for time representing the time point j. This term evaluates how the log odds change over time.
- γ_i is the random effect for individual i, accounting for the unobserved heterogeneity among individuals. This term allows for the model to include individual-specific variations in the log odds of the outcome.
- ϵ_{ij} is the residual error term for individual i at time point j, capturing any unexplained variability in the log-odds of the outcome.

With the proposed algorithm, predicted values are carried out by implementing the following steps:

1. First, initial values of the binomial distribution's parameters μ_{ij}, η_{ij} *and* w_{ij} are computed starting from y_{ij}.The value μ_{ij} is the expected value of Y_{ij}; η_{ij} is the linear predictor, the logit function of the model's parameters and predictors; w_{ij} represents weights applied to the observations, computed based on the variance of the expected value concerning the linear predictor.
2. Then the RF model is estimated on the given set of covariates.
3. The residuals are computed as the difference between the mortality outcome and the gained predicted values.
4. Afterward, the mixed model is estimated with the residuals computed in the previous step. The convergence of this model is checked through the change in the absolute value of the log-likelihood.
5. Then the response variable is updated by subtracting the estimated Random Effects (RE).
6. The initial values $Y_{ij}, \mu_{ij}, \eta_{ij}$ *and* w_{ij} are updated. The maximum changes in the absolute value of η_{ij} are monitored for the main loop's convergence.
7. Finally, the predicted values gained from the two models are extracted and presented as predicted probabilities of the binary outcome.

These steps are summarized in Figure S1.

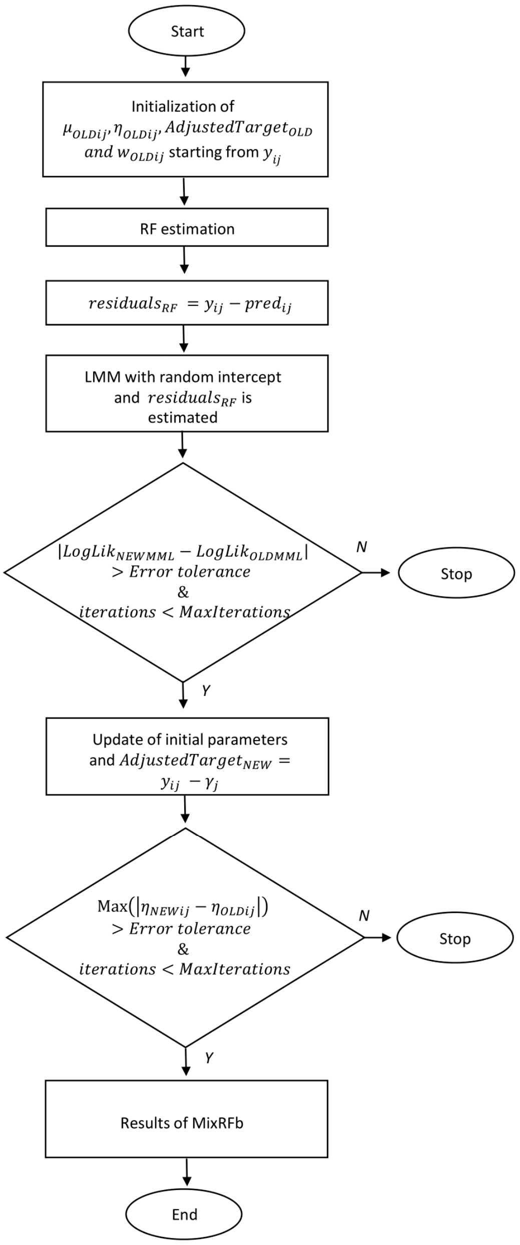

Figure S1 Flowchart of MixRFb algorithm

Table S1 Longitudinal clinical characteristics of ICU patients.

		**Median**	**[IQR]**		
**Variables**	**Day 1**	**Day 2**	**Day 3**	**Day 4**	**Day 5**
**RDW** (fL)	15.0 [14.0-16.7]	15.0 [14.0-17.0]	15.1 [14.0-17.0]	15.3 [14.0-17.0]	15.6 [14.3-17.0]
**Urea** (mmol/L)	46.0 [30.0-77.0]	46.0 [29.0-77.0]	46.0 [30.5-80.5]	47.5 [32.0-80.0]	49.0 [32.0-77.5]
**Procalcitonin** (ng/ml)	2.01 [0.69-8.32]	3.00 [0.71-13.5]	1.00 [0.50-6.20]	1.40 [0.38-3.82]	1.27 [0.56-4.32]

**PCR** (mg/ml)	16.0 [7.78-27.5]	22.1 [15.5-29.5]	21.6 [13.0-26.5]	13.1 [6.32-22.6]	11.1 [7.65-22.0]
**pH**	7.39 [7.34-7.43]	7.40 [7.32-7.43]	7.41 [7.31-7.45]	7.42 [7.32-7.45]	7.42 [7.31-7.46]
**PaO2** (mmHg)	109 [86.0-137]	94.3 [79.4-110]	93.0 [81.1-111]	92.0 [79.0-115]	88.5 [79.0-117]
**Lactate** (mmol/L)	1.30 [1.00-2.10]	1.20 [0.00-1.50]	1.20 [0.00-1.50]	1.20 [0.00-1.42]	1.10 [0.25-1.50]

RDW = Red blood cell Distribution Width; PCR = Polymerase Chain Reaction; PaO2 = Arterial Oxygen Pressure

Flow of Participants Through the Study

Study Design and Participant Flow

- Recruitment: Patients were identified upon admission to the Intensive Care Unit (ICU) of the University Hospital of Ferrara between August 2016 and December 2017.
- Eligibility Assessment: Each patient's eligibility was assessed based on specific inclusion criteria, primarily requiring an ICU stay of at least 48 hours. Patients who did not meet this criterion or who opted out were excluded from the study.
- Enrollment: A total of 300 patients were initially considered for inclusion. After applying the eligibility criteria, 286 patients were enrolled in the study.
- Follow-up: The follow-up period involved tracking patients' outcomes during their ICU stay. All enrolled patients were followed up for the duration of their ICU stay, with the primary outcome being in-hospital mortality.
- Outcome Assessment: Out of the 286 enrolled patients, 207 survived their ICU stay, while 79 succumbed to their conditions.

Summary of Follow-up Time

The follow-up time for each participant was equivalent to their length of stay in the ICU. The average stay was approximately 5 days, though this varied significantly depending on patient condition and outcomes. The longest stay recorded was 22 days.

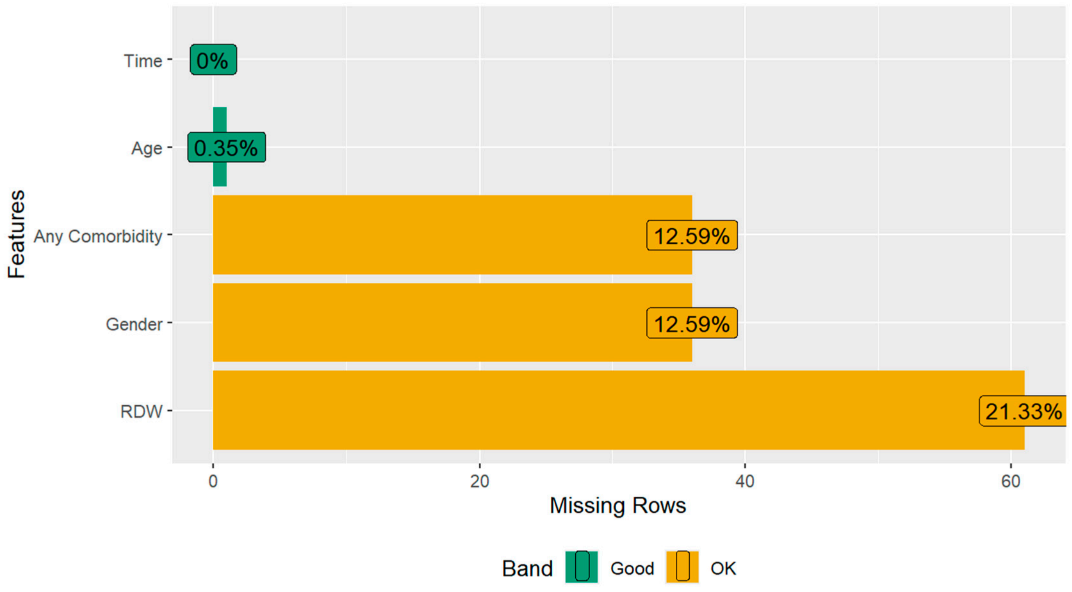

Figure S2 Missing Data Plot. Proportion of missing data for features in the dataset, categorized by the total percentage of missing rows for each feature. The y-axis lists the features, relabeled as "Time," "Age," "Any Comorbidity," "Gender," and "RDW" for clarity. The x-axis shows the number of missing rows, with labels indicating the corresponding percentage of missing values for each feature. Features are grouped into bands based on the percentage of missing data, with "Good" (green) representing features with minimal missing data and "OK" (orange) for features with a higher proportion of missing values.

Interaction Analysis

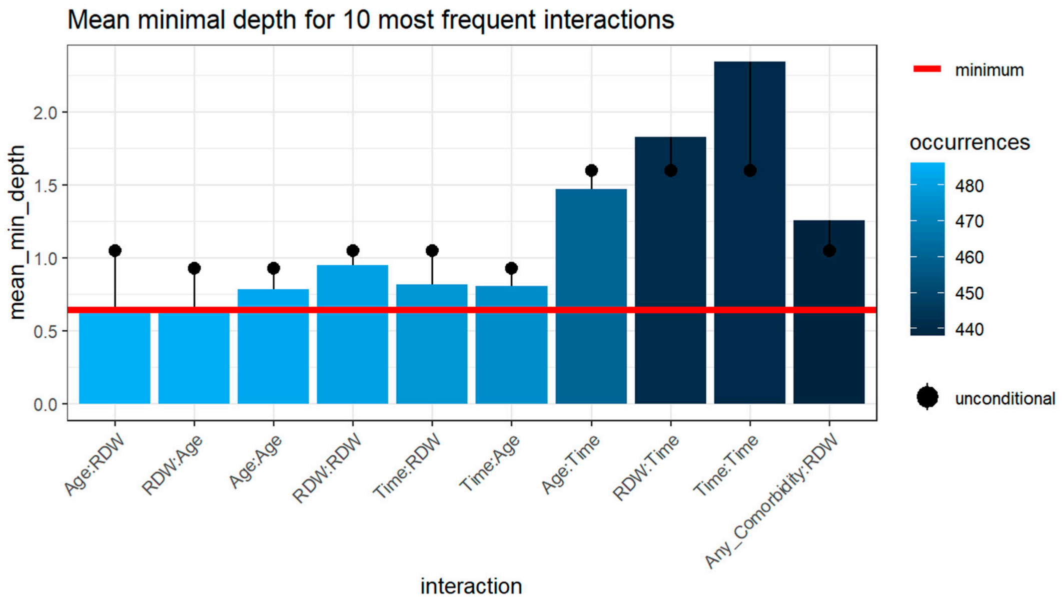

Figure S3 Mean Minimal Depth interaction plot. Mean minimal depth for the ten most frequent variable interactions identified in the random forest model. The x-axis represents variable interactions, while the y-axis indicates the mean minimal depth, which measures how early a variable interaction is utilized in the decision trees. Lower minimal depth reflects higher importance, as the interaction is used earlier in the model's decision-making process. The red horizontal line represents a predefined threshold for minimal depth, with interactions below the line considered highly significant. The bars are colored to indicate the frequency of the interaction's occurrence across the forest, with darker colors denoting higher frequencies. Black dots represent the unconditional importance of each interaction, calculated as the average impact of the interaction across the entire forest, independent of specific

conditions. Error bars around the black dots indicate variability in unconditional importance, providing insight into the stability and consistency of each interaction's significance.

The analysis of minimal depth provides insights into the importance of variables and their interactions in a random forest model. Minimal depth measures how early a variable or interaction appears in the decision trees, with lower minimal depth indicating higher importance. In this study, interactions such as Age:RDW and RDW:Age demonstrated low minimal depth, highlighting their significant role in the model's predictions. These interactions suggest a strong relationship between age and red cell distribution width (RDW) that may vary across age groups, emphasizing their clinical relevance.

The presence of interactions labeled as Age:RDW and RDW:Age reflects the algorithm's process, as random forests prioritize splits differently in various trees. Although these labels appear distinct, they represent the same underlying relationship—how age and RDW interact to influence outcomes.

Additionally, the "unconditional importance," represented by black dots and error bars, provides a complementary perspective. It measures the interaction's overall contribution across the forest, independent of specific conditions. Stable, low unconditional importance values (with small error bars) indicate consistently impactful interactions across trees, as observed with Age:RDW. In contrast, interactions like Time:Time had higher minimal depths and wider error bars, suggesting limited and variable importance.

This analysis underscores nonlinear role of critical predictors like age and RDW in the model. By identifying key interactions with both minimal depth and unconditional importance.

Algorithm's code

```
MixRFb <- function(y, x, random, data, initialRandomEffects = 0,lev=("Yes"),

              ErrorTolerance = 0.001, MaxIterations = 1,

              ErrorTolerance0 = 0.001, MaxIterations0 = 1, verbose = FALSE) {


 # Condition that indicates the loop has not converged or run out of iterations

 ContinueCondition0 <- TRUE

 iterations0 <- 0


 # y<-as.numeric(as.character(y))

 # y<-ifelse(y==lev,1,0)


 # Get initial values

 mu <- rep(mean(y), length(y))
```

```
eta <- log(mu / (1 - mu))
lp <- eta + (y - mu) / (mu * (1 - mu))
weights <- mu * (1 - mu)

AdjustedTarget <- lp - initialRandomEffects

f1 <- as.formula(paste0('AdjustedTarget ~ ',paste(colnames(x),collapse = "+")))
f0 <- as.formula(paste0('resi ~ -1 + ', random))

# mimic randomForest's mtry
ncol <- ncol(x)
mtry <- if (!is.null(y) && !is.factor(y))
  max(floor(ncol / 3), 1) else floor(sqrt(ncol))
# mtry=params$mtry

oldLogLik <- oldEta <- -Inf

# PQL - PENALIZED QUASI-LIKELIHOOD
while (ContinueCondition0) {

  iterations0 <- iterations0 + 1
  iterations <- 0
  ContinueCondition <- TRUE

  # random forest + lmer
  while (ContinueCondition) {

    iterations <- iterations + 1

    # random Forest
    data$AdjustedTarget <- AdjustedTarget
    rf <- randomForest(f1, data = data, weights=weights)
```

```
  # y - X*beta (out-of-bag prediction)
  pred <- predict(rf, OOB = T)

  resi <-  lp - pred

  ## Estimate New Random Effects and Errors using lmer
  lmefit <- lmer(f0, data = data, weights = weights)

  # check convergence
  LogLik <- as.numeric(logLik(lmefit))

  ContinueCondition <- (abs(LogLik - oldLogLik) > ErrorTolerance & iterations < MaxIterations)
  oldLogLik <- LogLik

  # Extract (the only) random effects part to make the new adjusted target
  AllEffects <- predict(lmefit)

  #  y-Zb
  AdjustedTarget <- lp - AllEffects

  # monitor the change the of logLikelihood
  if (verbose) print(c(iterations0, iterations, LogLik))
}

eta <- pred + AllEffects
mu <- 1 / (1 + exp(-eta))
lp <- eta + (y - mu) / (mu * (1 - mu))
AdjustedTarget <- lp - AllEffects
weights <- as.vector(mu * (1 - mu))

print(c(iter = iterations0, maxEtaChange = max(abs(eta - oldEta))))

ContinueCondition0 <- (max(abs(eta - oldEta)) > ErrorTolerance0 & iterations0 < MaxIterations0)
```

```
    oldEta <- eta
  }

  result <- list(forest = rf, MixedModel = lmefit, RandomEffects = ranef(lmefit),
                 IterationsUsed = iterations0)

  return(result)
}

predict.MixRFb <- function(object, newdata, EstimateRE = FALSE, as_probabilities = F) {

  forestPrediction <- predict(object$forest, newdata = newdata, OOB = T)

  # If not estimating random effects, just use the forest for prediction.
  if (!EstimateRE) {
    if (as_probabilities) {
      return(1 / (1 + exp(-forestPrediction)))
    } else {
      return(forestPrediction)
    }
  }

  RandomEffects <- predict(object$MixedModel, newdata = newdata, allow.new.levels = TRUE)

  completePrediction = forestPrediction + RandomEffects

  if (as_probabilities) {
    return(1 / (1 + exp(-completePrediction)))
  } else {
    return(completePrediction)
  }
}
```

```
prob.MixRFb <- function(object, newdata, EstimateRE = FALSE, as_probabilities = TRUE) {

  forestPrediction <- predict(object$forest, newdata = newdata, OOB = T)

  # If not estimating random effects, just use the forest for prediction.
  if (!EstimateRE) {
    if (as_probabilities) {
      return(1 / (1 + exp(-forestPrediction)))
    } else {
      return(forestPrediction)
    }
  }

  RandomEffects <- predict(object$MixedModel, newdata = newdata, allow.new.levels = TRUE)

  completePrediction = forestPrediction + RandomEffects

  if (as_probabilities) {
    return(1 / (1 + exp(-completePrediction)))
  } else {
    return(completePrediction)
  }
}
```

References

57. Hothorn, T.; Hornik, K.; Zeileis, A. Unbiased Recursive Partitioning: A Conditional Inference Framework. *J. Comput. Graph. Stat.* **2006**, *15*, 651–674, doi:10.1198/106186006X133933.
58. Xia, R. Comparison of Random Forests and Cforest: Variable Importance Measures and Prediction Accuracies. **2009**, doi:10.26076/74BD-59E1.
